# Vocation and avocation: leisure activities correlate with professional engagement, but not burnout, in a cross-sectional survey of UK doctors

**DOI:** 10.1186/1741-7015-9-100

**Published:** 2011-08-30

**Authors:** I C McManus, Hallgeir Jonvik, Peter Richards, Elisabeth Paice

**Affiliations:** 1 Academic Centre for Medical Education, Division of Medical Education, University College London, Gower Street, London WC1E 6BT, UK; 2 Division of Psychology and Language Sciences, University College London, Gower Street, London WC1E 6BT, UK; 3 Hughes Hall, Cambridge CB1 2EW, UK; 4 London Deanery, Stewart House, 32 Russell Square, London WC1B 5DN, UK

## Abstract

**Background:**

Sir William Osler suggested in 1899 that avocations (leisure activities) in doctors are related to an increased sense of vocation (professional engagement) and a decreased level of burnout. This study evaluated those claims in a large group of doctors practicing in the UK while taking into account a wide range of background variables.

**Methods:**

A follow-up questionnaire was sent to 4,457 UK-qualified doctors who had been included in four previous studies of medical school selection and training, beginning in 1980, 1985, 1990 and 1989/1991. A total of 2,845 (63.8%) doctors returned the questionnaire. Questions particularly asked about work engagement, satisfaction with medicine as a career, and personal achievement (Vocation/engagement), stress, emotional exhaustion, and depersonalization (BurnedOut), and 29 different leisure activities (Avocation/Leisure), as well as questions on personality, empathy, work experience, and demography.

**Results:**

Doctors reporting more Avocation/Leisure activities tended to be women, to have older children, to be less surface-rational, more extravert, more open to experience, less agreeable, and to fantasize more. Doctors who were more BurnedOut tended to be men, to be more sleep-deprived, to report a greater workload and less choice and independence in their work, to have higher neuroticism, lower extraversion and lower agreeableness scores, and to have lower self-esteem. In contrast, doctors with a greater sense of Vocation/engagement, tended to see more patients, to have greater choice and independence at work, to have a deep approach to work, to have a more supportive-receptive work environment, to be more extravert and more conscientious, and to report greater self-esteem.

Avocation/Leisure activities correlated significantly with Vocation/engagement, even after taking into account 25 background variables describing demography, work, and personality, whereas BurnedOut showed no significant correlation with Avocation/Leisure activities. Popular Culture and High Culture did not differ in their influence on Vocation/engagement, although there was a suggestion that Depersonalization was correlated with more interest in Popular Culture and less interest in High Culture.

**Conclusion:**

In this cross-sectional study there is evidence, even after taking into account a wide range of individual difference measures, that doctors with greater Avocation/Leisure activities also have a greater sense of Vocation/Engagement. In contrast, being BurnedOut did not relate to Avocation/Leisure activities (but did relate to many other measures). Osler was probably correct in recommending to doctors that, 'While medicine is to be your vocation, or calling, see to it that you also have an avocation'.

## Background

Doctors are often described as having a 'vocation' ... a calling ...which makes medical practice less a job and more a way of life. In modern terms, 'vocation' might perhaps be better described as 'work engagement' [1,2], 'efficacy' or a sense of 'personal accomplishment' [3].

Medicine can, however, also be very stressful, so that the sense of vocation becomes overwhelmed. The physician, Sir William Osler, described in 1905 [4], how it is,

'...only zeal, a fiery passion, [which] keeps the flame alive, smothered as it is so apt to be by the dust and ashes of the daily routine.' (p.418)

Nowadays that smothering of the flame would probably be called burnout [3], a problem that can affect all professionals. Not all doctors however suffer from burnout, in large part because doctors, like all people, differ, in personality, background, working environment, and so on [5]. The result, as Osler said, is that:

'To each one of you the practice of medicine will be very much as you make it ... to one a worry, a care, a perpetual annoyance; to another, a daily joy and a life of as much happiness and usefulness as can well fall to the lot of man.' (p.423)

Osler had earlier, in 1899, provided an important piece of advice to young doctors on how to avoid burnout, suggesting that as well as having a vocation, doctors should also have an avocation, saying:

'While medicine is to be your vocation, or calling, see to it that you also have an avocation... some intellectual pastime which may serve to keep you in touch with the world of art, of science, or of letters. Begin at once the cultivation of some interest other than the purely professional. The difficulty is in a selection and the choice will be different according to your tastes and training. No matter what it is B but have an outside hobby.' (p.204)

Lest it be thought, in the 21^st ^century, to be inappropriate to take seriously the ideas of someone writing more than a century earlier, it should briefly be emphasised that Osler is still frequently quoted in the medical literature (and for instance, Osler's *Aequanimitas*, alone, according to *ISI Web of Knowledge*, was cited 178 times between 2000 and 2011, and 111 times in the decade before that).

### Literature search

A search of MEDLINE (7^th ^April 2011) retrieved 476,849 studies containing 'doctor* OR physician*', and 12,729 studies containing 'avocation OR leisure* OR hobby OR hobbies', with 733 studies referring to both. The vast majority of these studies were, however, not relevant to the present study. A large number were on the hobbies of deceased doctors, sometimes historical, were on possible hobbies for doctors (for example, photography, music, succulent collecting or numismatics), or on how leisure activities affect patients (for example, in diabetes, or as a risk factor for breast cancer), on disease presentations as a result of leisure activities in the general population (for example, vertebral artery dissection after a roller coaster ride, Lyme Disease in people with outdoor leisure activities, or injuries acquired during campanology), and on one occasion on disease resulting from the leisure activities of doctors themselves, in a study of tennis-elbow [6]. None were relevant to the purposes of the present analysis, although it might be worth mentioning one somewhat contrarian paper which emphasized the sometime dangers to health resulting from leisure and leisure activities, and suggested that leisure activities are not therefore an unmitigated good [7].

Many other studies addressed questions about the leisure activities of doctors, but consisted either of paeans to the need for doctors to relax and look after themselves more or to be more involved in leisure, or were descriptions of the leisure activities of particular doctors [8-12]. A further set of articles assessed job and life satisfaction in doctors, and often but not always, reported dissatisfaction with time available for leisure activities [13-26], with sufficient time for leisure activities correlating with quality of life measures [27], and not having hobbies being a risk factor for hypertension in doctors [28]. Some studies also reported higher life satisfaction, including participation in hobbies and leisure activities, in retired doctors [29].

Other studies have reported that opportunities for leisure activities impact on decisions by doctors on choice of speciality and choice of practice location [30-34] or the timing of retirement [35]. One study reported how demands of doctors for more leisure time themselves impacted upon service provision [36].

A few studies looked at leisure activities in relation to formal measures of stress or burnout, and reported that having a hobby correlated with lower levels of burnout in general [37], with emotional exhaustion [38,39] or with job stress [40], although sometimes there was no correlation with burnout [41].

A frequent comment in studies, which typically followed the line described earlier by Osler, was of the beneficial role of increasing leisure activities, shown most clearly in the sub-title of one article, which said, 'Hobbies relieve stress and allow self expression' [42]. A similar claim is made in a study of burnout that, '...enriching leisure activities ... [is one of several] important measures to preventing burnout' [43], and hobbies were regarded by specialists in palliative medicine as an important way to prevent burnout [44], as well as a way of preventing 'physician disability'" [45]. An extension of the claim is that, 'more leisure and unstructured contemplation of the humanities help physicians to cherish empathy' [46] (and there is a recurrent belief, rarely substantiated with evidence, that the humanities nurture humane behaviour in doctors specifically, and in general [47]). Few studies have considered the specific type of the leisure activities, although one study specifically looked at cultural activities, and in comparison with a control, university-educated, population found less time watching television, more time reading non-medical books, and more time devoted to music [48]. Comment has also been made upon an apparent tendency for doctors to be proficient in a range of non-medical areas, both as talented amateurs [49], and also the extent that what was once an avocation becomes a vocation, with distinction achieved outside medicine [50].

This review of the literature should perhaps conclude with some citations of Osler himself, particularly to show how his ideas are still being invoked. In 1990, Richard E. Clark, in his presidential address to the Southern Thoracic Surgical Association, cited Osler as follows [51]:

'No man is really happy or safe without a hobby, and it makes precious little difference what the outside interest may be - - botany, beetles or butterflies, roses, tulips or irises, fishing, mountaineering or antiquities - - anything will do so long as he straddles a hobby and rides it hard' [52].

The quotation was also used in a paper by a dermatologist, in 1998 [53], and then was referred to again, in the 2002 Presidential Lecture of the Southern Thoracic Surgical Association [54], where it was followed by a comment that, 'The complete cardiothoracic surgeon should have a hobby outside of medicine. ... It may be photography, literature or golf, but there is something that each of us needs: a diversion outside of the laboratory and operating theater'. (p.7).

### Burnout, stress and engagement

In conceptualizing the beneficial effects of leisure, writers often refer to the interlinked concepts of burnout, stress and job satisfaction, and in recent years, the idea of engagement has also come to be used in explanatory ways. The literature on each of these is huge, and no attempt will be made at completeness.

### Burnout

Osler, we have suggested, in referring to how an enthusiasm for medicine could be 'smothered ...by the dust and ashes of the daily routine... may have been referring to what is now called 'burnout', a term which apparently was first used as a technical term in 1975 [55]. In a series of studies since 1978 [56], the concept of burnout has been explored, conceptualized and measured by Maslach, with a key methodological innovation being the development of the Maslach Burnout Inventory (MBI) [57], with its separate subscales of Emotional Exhaustion (EE), Depersonalization (DP), and Personal Accomplishment (PA), with high EE and DP and low PA indicating burnout. The precise relationship between EE, DP and PA is unclear, and in particular whether they are measuring related or unrelated concepts. In a longitudinal study [58] we have found that the causal relationships between the subscales are different, suggesting that they are conceptually distinct. A key theoretical point is made by Maslach *et al*. [3], when they say, 'Maslach and Leiter (1997) rephrased burnout as an erosion of engagement with the job. What started out as important, meaningful and challenging work becomes unpleasant, unfulfilling and meaningless. Energy turns into exhaustion, involvement turns into cynicism, and efficacy turns into ineffectiveness. Accordingly, engagement is characterized by energy, involvement and efficacy -- the direct opposites of the three burnout dimensions' (p.416). The crucial point here is that engagement is seen as the polar opposite of burnout.

### Stress

Stress is a complex term [59,60], which has been much studied in health professionals [61-63]. Many different measures are available, but a popular one, which we use here, is the General Health Questionnaire (GHQ) [64] which has been used in a range of studies of doctors [65-67]. It should also be emphasized that stress can be seen as conceptually distinct from burnout, stress resulting from work stressors, whereas burnout originates in a lack of sense or purpose or importance for work [68].

### Job satisfaction

Job satisfaction is frequently measured in studies but rarely is well conceptualized, or even measured consistently, and it can mean many things. In our studies we have used the temporally-anchored response scale of the MBI ('every day', 'a few times a week', once a week' and so on), and included additional items asking about how often a doctor reflects on the satisfaction they get from being a doctor, thinks of giving up medicine for another career, and regrets their decision to become a doctor (the latter two being reverse scored) [69]. A rare study of the relationships of satisfaction to other measures found that both stress and burnout correlated with satisfaction, although the relationship was stronger with burnout [68].

### Engagement

Interest in work engagement has emerged only relatively recently [2], and in particular has been helped by the development of the Utrecht Work Engagement Scale (UWES) [1]. Conceptually work engagement appears to be distinct from burnout [70], and that is supported both by the lack of a strong correlation between them (so that it is possible for individuals to have very low burnout scales, but nevertheless not to be engaged [71,72], and also by them having different personality correlates [73]. Engagement can be seen as a positive condition compared with the negative condition of burnout, with the two arranged orthogonally (meaning it is possible to be both burned out and engaged). When Osler referred to the 'zeal, a fiery passion', he may have been referring to what now is conceptualized as 'engagement'.

### The present study

This study set out to investigate a number of separate but related aspects concerning the leisure activities of doctors and their relation to stress and burnout. In particular we wished:

To assess the widely held intuition, most venerably put forward by Osler, that the non-medical activities of doctors (their avocations) are related to their involvement and commitment to their daily professional activities (their vocation).

As well as measuring a wide-range of leisure activities, including those which may or may not be described as high-culture, we also assess the modern concepts of stress, burnout, work engagement and career satisfaction, in order to take apart the relationship more clearly.

Given that stress, burnout and engagement are known to relate to individual difference measures, we assess to what extent any relationships with leisure activities are themselves secondary to differences in personality (assessed using the standard Big Five conceptualization). It may be that, for example, burnout and leisure activities are intercorrelated because the same personality characteristic predisposes to burnout and a lack of leisure activities. Perhaps it is not avocations *per se *that are important, but that those individuals with more avocations also have a personality that encourages a sense of vocation.

We have also taken into account a large number of background variables, of work load, of work experience, of other individual difference measures and so on, in order to assess whether they may account for any correlations of leisure activities with stress, burnout, satisfaction and engagement. Individual difference measures of personality, empathy, masculinity and self-esteem were included as it was thought, on theoretical grounds, that they may relate either to leisure activities or to stress, burnout, engagement and satisfaction.

The study itself, for present purposes, is a cross-sectional study of a large group of doctors surveyed in 2009. It should be noted that these same doctors are part of several different longitudinal cohort studies, although the longitudinal aspects of the studies will not be considered here. Likewise, a number of other measures were collected above those reported here, which are restricted to the testing of Osler's hypothesis.

## Methods

In March 2009 a follow-up was carried out of several large groups of doctors who had previously taken part in longitudinal, prospective studies of medical student selection, training, and postgraduate education, and who had originally applied for entry to medical school in 1981 [74,75], 1986 [76,77], 1991 [78,79], and 1990/1992 [80], and had been followed up on a number of occasions [5,69,75,77,79,81]. The postal questionnaire, which consisted of one sheet of folded A3 paper (four sides of A4) containing a range of questions, was sent with a reply-paid envelope. After the initial mailing, three reminders were sent to non-respondents. Addresses of doctors were provided by the General Medical Council (GMC). Of 4,457 questionnaires not returned by the Post Office, 2,845 were returned satisfactorily, giving a response rate of 63.8%. Not all doctors provided answers to all questionnaire items, and mean substitution for missing values has been used where appropriate.

### Measures

A range of different questionnaire measures were used in the study. The questionnaire used in the current analyses can be found in the Additional File, with the exception of one part which has been removed for copyright reasons. Q.n refers to question 'n' of the questionnaire.

### Leisure activities

Participation in 29 leisure activities was assessed in Q.21 of the questionnaire, using temporally anchored scales to assess the extent of each activity; 17 and 20 of the activities had been asked about in previous studies [82,83], and the remainder were new for this study, the range of topics being extended to make it more appropriate for doctors, and to cover topics which, due to space and other constraints, had been omitted in the original.

### Stress

As in our previous studies, stress was assessed in Q.20 using the twelve-item version of the GHQ, with standard wording and 0-1-2-3 scoring.

### Burnout

As in previous studies, we used an abbreviated, nine-item version of the Maslach Burnout Inventory (aMBI), Q.16, with three questions each for assessing EE, DP and PA [5,58,84]. Scores for EE (items 4,5,9), DP (2,7,11) and PA (1,8,12) were calculated by summing the individual items.

### Engagement

We have not assessed Work Engagement previously, and in this study we used a very abbreviated, three-item version of the Utrecht Work Engagement Scale (aUWES) {34346, 34531), with items 1, 6 and 10 from the original UWES scale. The items were in Q.16, with item 6 from the Vigor scale, item 10 from the Absorption scale, and item 3 from the Dedication scale. Items were intermixed with the Satisfaction and aMBI items, and used the seven-point temporally anchored scale originally devised for the aMBI. For an overall score, the three items were summed.

### Job Satisfaction

The three items measuring job satisfaction are the same as those used in previous studies [69], Q.16, items 13,14,15. For an overall score Satisfaction (SATN), the three items were summed, with 13 and 15 being reverse scored.

### Work variables

A number of measures were used to assess aspects of work and the work environment.

### Type of work

The UK's General Medical Council register of doctors (LRMP: List of Registered Medical Practitioners) was used to determine whether or not a doctor was on the Specialist Register or on the General Practice Register.

### Work load

Q.5 asked doctors how many patients they saw in a typical week in six different situations. A simple sum of the encounters was used as a measure of number of patients seen. Q.6 assessed the extent to which sleep deprivation occurred because of typical working patterns.

### Approach to Work Questionnaire (AWQ) and Workplace Climate Questionnaire (WCQ)

Q.15 used an abbreviated version of these questionnaires [85-87], which had also been used in an abbreviated form in a previous study [5]. The abbreviated AWQ (aAWQ) had two items on each of the three subscales (Deep approach to work, items 3 and 6; Surface-rational, items 2 and 5; and Surface-Disorganized, items 1 and 4), and the abbreviated WCQ (aWCQ) also had two items on each of the three sub-scales (Choice and Independence, items 7 and 9; Workload, items 10 and 12; Supportive-Receptive, items 8 and 11). Scores on each subscale were derived by summing items.

### Individual difference and personality measures

Individual differences were measured, as in previous studies, by using abbreviated versions of various scales.

### Personality

Q.17, items 1 to 15 (not shown in Additional File 1) contained a fifteen-item abbreviated version of the NEO measure of the Big Five personality dimensions [88], which has been used in other studies on a range of topics [82,83,89,90].

### Empathy

Q.18 contained a twelve-item version of the Inter-personal Reactivity Index [91,92], which has four sub-scales (Fantasy: items 1, 6 and 11; Perspective-taking, items 2, 5 and 12; Empathic concern, items 3, 7 and 10; and Personal Distress, items 4, 8 and 9). We have used these scales in several previous studies of these cohorts, but have not previously published results using them.

### Masculinity-Femininity

Q.19 contained an eight-item abbreviated version of Spence and Helmreich's Personal Attributes Questionnaire, which is a measure of masculinity and femininity [93]. Responses were scored as a single scale, with positive values indicating masculinity and negative values indicating femininity. Items 2, 3, 5 and 8 were reverse scored.

### Self-esteem

Q.18, items 16, 17, 18 and 19, were four items from the Rosenberg Self-Esteem scale [94]. A single scale was constructed by summing the items, with items 17 and 19 being reverse scored.

### Demographic variables

Year of qualification as a doctor and sex were obtained from the LRMP. Respondents also described the number of children they had, and their ages, and from this we derived two summary measures: Number of Children and Age of Youngest Child, the latter being felt to be most useful as a proxy for the extent to which having children imposed on other activities.

### Statistical methods

Statistical analysis used SPSS 13.0. Where indicated, missing values were replaced using mean substitution. Calculation of significance is always difficult in any study with a large number of variables, particularly if there is a large number of participants, as there is a risk on the one hand of type I errors, due to repeated significance testing, and on the other hand of effects being highly significant, but effect sizes being small. There is no simple solution to this, particularly if one believes that complex social phenomena are likely to have multiple causes, rather than a single one, so that many variables need to be evaluated in order to get a suitably rich analysis of underlying processes (for an elegant discussion of these issues see Sherman and Funder [95]). In this paper, we report significance levels as they are (that is, without Bonferroni or other correction), and we indicate those which are conventionally significant (and that is of help to those attempting to replicate effects). In interpreting results, particularly when, for instance, many background variables are entered into analyses on an *a posteriori *basis, we mostly restrict ourselves to discussing those which reach a level of *P *< .001, and we also carry out portmanteau tests, to check that sets of variables are jointly significant as a set, since such tests are far less subject to alpha inflation. A few of our tests are *a priori*, in particular of links between leisure activities and stress/burnout/engagement/satisfaction levels. For those tests we consider results which are significant in a conventional sense (that is, *P *< .05). Finally we emphasize that the ultimate test of significance, in any study, is that effects which are 'significant' in one study, replicate in another. If other researchers are uncertain about the true significance of results then a partial obligation is upon them to replicate, particularly when, as in this case, there are no other comparable studies, either in breadth of measures, or in the ability to look at these particular issues.

### Ethics

This research study was discussed with the Chair of the UCL Ethics Committee, who stated that the project was exempt from requiring formal ethics permission (see http://ethics.grad.ucl.ac.uk/exemptions.php).

## Results

The modal year of qualification as a doctor in the 1981, 1986, 1991 and 1990/92 cohorts was 1986, 1991, 1996 and 1996, the doctors having been qualified for 23, 18, 13 and 13 years at the time of this study. Their mean ages were 46.9, 41.9, 37.0 and 36.9 years (SDs 3.23, 1.87, 1.84 and 2.00). The proportion of female respondents differed between the cohorts (1981: 43.7%, 1986: 48.6%, 1991: 56.7% 1990/92: 55.2%).

### Avocation/Leisure activities

Table 1 shows how much the doctors took part in the 29 activities. There are correlations with sex and years qualified on many of the individual activities. Factor analysis of the activities showed a large first eigenvalue, and therefore for the main analyses a single factor score was extracted based on all 29 activities, with an alpha reliability of 0.747. This factor we call Avocation/Leisure. Table 2 shows the loadings, a ) based on extraction of a single factor; and b) extraction of two oblique factors, which are labelled High Culture and Popular Culture, with the oblique factors correlating 0.183. Absolute factor loadings >.4 are shown in bold, and those less than 0.1 are left blank. The eigenvalues of the correlation matrix were: 3.97, 2.16, 1.63, 1.46, 1.39, 1.28, 1.18, 1.12, 1.02, .97, .96, .91, .88, .85, .82, .78, .75, .74, .69, .67, .65, .62, .60, .57, .56, .51, .46, .44 and .38, and although a scree-plot suggested one main factor, it could be argued that there are two main factors present. We therefore extracted two oblique factors using Direct Oblimin in SPSS (an oblique rotation was used since there was no theoretical reason to believe that two factors had to be orthogonal, and it was more than possible that they were correlated). The two factors correlated positively with a value of .183; their loadings are shown in table 2. The first factor consists broadly of what can be called 'High Culture' (mainly consisting of theatre, museums, classical music, art, literature, and so on), whereas the second factor we describe as 'Popular Culture' (consisting principally of television, internet, sport, popular music, watching DVDs, and so on). Although the main interest of the rest of the study will concern Avocation/Leisure activities, the High Culture and Popular Culture scores will also be analyzed, particularly since there is a suggestion in the literature (see the Introduction) that High Culture may have different effects than Popular Culture.

**Table 1:** Frequencies of a range of leisure activities, and correlations with a) being male, b) number of years qualified, c) *BurnedOut*, and d) *Vocation/Engagement*

**How often do you?**	**Every day**	**A few times** ** a week**	**Once** ** a week**	**A few times** ** a month**	**Once a month** ** or less**	**A few times** ** a year**	**Never**	**N**	**Male**	**Years** **qualified**	**Burned** **Out**	**Vocation/** **Engagement**
	
Listen to popular music	44.0%	35.7%	8.4%	6.1%	3.2%	1.6%	1.0%	2826	.018	-.034	-.099***	.079***
	
Listen to classical music	9.1%	24.3%	14.8%	15.7%	12.4%	14.7%	9.0%	2828	-.063***	.069***	-.032	.057**
	
Go to pop concerts/discos	-	-	.5%	1.7%	-8.0%	41.5%	48.3%	2829	.117***	-.036	-.007	.058**
	
Go to classical music concerts/opera	-	-	.1%	.8%	4.4%	38.7%	55.9%	2824	-.040*	.081***	-.023	.061**
	
Play a musical instrument	2.3%	6.8%	3.4%	6.5%	7.6%	14.8%	58.6%	2833	.003	.087***	-.022	.036
	
Go to museums or art galleries	-	.1%	.5%	4.9%	18.3%	59.4%	16.7%	2825	-.018	-.004	-.038	.055**
	
Read about art in newspapers, magazines or books	3.6%	7.4%	13.1%	13.8%	15.1%	23.7%	23.2%	2826	.045*	.080***	-.008	.082***
	
Draw, paint, sculpt or do other arts or crafts	.4%	3.1%	3.6%	7.0%	9.1%	20.8%	56.1%	2824	-.202***	-.037	-.043*	.056**
	
Photography	.8%	5.9%	8.7%	22.0%	16.8%	24.0%	21.8%	2839	.054**	-.028	-.080***	.095***
	
Read a novel	20.8%	17.2%	5.9%	12.4%	15.5%	21.0%	7.2%	2833	-.184***	.029	-.041*	.078***
	
Read non-fiction books (not for work or study)	7.3%	10.9%	5.6%	13.1%	17.0%	29.8%	16.3%	2828	.119***	.028	-.002	.105***
	
Read poetry	.2%	.6%	1.5%	4.0%	6.7%	25.1%	61.9%	2825	-.113***	.062***	.001	.094***
	
Write poetry, fiction or other literature (not for work)	.3%	.9%	.4%	1.0%	2.5%	6.3%	88.8%	2813	.029	.013	.038	.027
	
Go to the cinema	-	.1%	1.8%	9.6%	27.8%	52.0%	8.7%	2830	.005	.007	-.004	.0994***
	
Go to the theatre (plays/musicals, etc)	.1%	-	.1%	1.7%	12.8%	64.8%	20.5%	2830	-.059**	.075***	-.033	.105***
	
Acting or otherwise taking part in theatre	.1%	.2%	.3%	.3%	.4%	2.4%	96.3%	2826	-.004	.018	.019	-.013
	
Watching classical or modern ballet/dance	-	.1%	.1%	.5%	1.8%	24.3%	73.3%	2829	-.171***	.031	-.057**	.054**
	
Dance (any form)	.3%	1.2%	2.5%	2.3%	4.6%	21.6%	67.6%	2816	-.140***	-.071***	-.064**	.079***
	
Play sport	4.0%	26.3%	13.5%	10.6%	10.5%	17.4%	17.7%	2816	.103***	.062**	-.072***	.114***
	
Watch sport	2.0%	15.3%	12.1%	15.1%	13.2%	21.3%	21.0%	2816	.360***	.092***	-.055**	.093***
	
Hike/Orienteer/Climb/Mountaineer/Ski etc.	.5%	3.7%	4.3%	9.0%	13.6%	40.9%	28.0%	2791	.033	.073***	-.011	.096***
	
Cook	42.8%	32.3%	7.9%	6.8%	3.8%	4.0%	2.5%	2808	-.379***	-.073***	-.062**	.006
	
Shop (for pleasure)	.5%	3.3%	8.1%	26.8%	32.3%	20.3%	8.7%	2813	.167***	.075***	.004	.072***
	
Spend time on hobbies (excluding above activities)	4.2%	17.6%	15.0%	19.6%	15.0%	15.9%	12.7%	2671	.126***	.011	-.063**	.140***
	
	*--------------------- Most days for --------------------*											
	
	*4+ hours*	*2-4 hours*	*1-2 hours*	*1 hour or less*	*2-3 times a week*	*Once a week*	*Less often*	*N*				
	
Watch television	1.6%	12.4%	34.8%	26.5%	14.8%	5.8%	4.2%	2816	.079***	-.063***	.077***	-.059***
	
Watch DVDs/videos/etc..	.3%	1.3%	5.7%	10.2%	11.0%	31.1%	40.4%	2794	.145***	-.049**	.042*	.012
	
Listen to radio	3.8%	11.7%	31.7%	36.2%	9.4%	3.6%	3.6%	2813	.003	.026	-.034	.038
	
Listen to podcasts	.1%	.5%	1.5%	4.0%	2.9%	6.0%	84.9%	2748	.127***	-.048*	.030	.015
	
Browse the internet (not for work)	1.3%	5.3%	17.9%	31.3%	26.1%	13.0%	5.1%	2812	.262***	-.119***	.061**	-.004

*: p < .05; **: p < .01; ***: p < .001.

**Table 2:** Factor analyses of leisure activities.

	**Single factor**	**Two oblique factors**	
		
		**"High Culture"**	**"Popular culture"**
	
Listen to popular music	0.277		**0.484**
	
Listen to classical music	**0.447**	**0.537**	-0.103
	
Go to pop concerts/discos	0.392	0.159	**0.465**
	
Go to classical music concerts/opera	**0.560**	**0.611**	
	
Play a musical instrument	0.321	**0.401**	-0.102
	
Go to museums or art galleries	**0.575**	**0.543**	0.131
	
Read about art in newspapers, magazines or books	**0.525**	**0.446**	0.208
	
Draw, paint, sculpt or do other arts or crafts	0.376	**0.441**	
	
Photography	0.285	0.198	0.192
	
Read a novel	**0.473**	**0.445**	0.111
	
Read non-fiction books (not for work or study)	**0.464**	0.376	0.215
	
Read poetry	**0.517**	**0.599**	
	
Write poetry, fiction or other literature (not for work)	0.328	0.334	
	
Go to the cinema	**0.462**	0.280	0.384
	
Go to the theatre (plays/musicals, etc)	**0.607**	**0.536**	0.204
	
Acting or otherwise taking part in theatre	0.211	0.249	
	
Watching classical or modern ballet/dance	**0.451**	**0.518**	
	
Dance (any form)	0.331	0.324	
	
Play sport	0.236		0.316
	
Watch sport		-0.251	**0.526**
	
Hike/Orienteer/Climb/Mountaineer/Ski etc.	0.268	0.217	0.124
	
Cook	0.258	0.292	
	
Shop (for pleasure)	0.314	0.127	0.374
	
Spend time on hobbies (excluding above activities)	0.317	0.152	0.334
	
Watch television		-0.361	**0.502**
	
Watch DVDs/videos/etc..	0.240		**0.459**
	
Listen to radio	0.211		0.363
	
Listen to podcasts	0.166		0.301
	
Browse the internet (not for work)	0.109	-0.195	**0.556**

a) loadings based on extraction of a single factor; and b) extraction of two oblique factors, which are labelled High Culture and Popular Culture. Absolute factor loadings >.4 are shown in bold, and those less than 0.1 are left blank. The oblique factors correlate 0.183.

### Correlates of Avocation/Leisure activities

Table 3 shows the regression of Avocation/Leisure, High Culture and Popular Culture on the 25 background variables. For each dependent variable (columns), standardised (beta) coefficients are shown, with, for comparison, simple Pearson correlations with each background variable also shown in square brackets within each cell. In these, and in all other regressions, the entire set of background variables is added simultaneously, the overall significance of the set is tested, and the significance levels shown in Table 3 are therefore of each variable, taking into account all of the other 24 variables already in the model, so that they are true effects of each measure (and are therefore conservative in nature). For each of the three leisure measures, the overall (portmanteau) effects are highly significant, with the set of background variables accounting for up to a quarter of the total variance (F(25,2813) = 33.54, *P *<< .001; 37.47, *P *<< .001 and 23.27, *P *<< .001, R = .479, .500, and .414, accounting for 23.0%, 25.0% and 17.1% of total variance, respectively). Correction for attenuation due to measurement error, suggests that the background variables explain 30.8% of the accountable variance in Avocation/Leisure. In assessing the significance of individual correlates of the scores in Table 2, with 25 background variables it should be remembered that if all background variables were independent, then with *P *< .05, *P *< .01 and *P *< .001 only 1.25, 0.25 and 0.025 correlates would be expected to be significant by chance alone.

**Table 3:** Beta coefficients from multiple regression for association of background variables with *Avocation/Leisure activities*, *High Culture*, *Popular Culture*, *Vocation/Engagement *and *Burned Out*.

	Avocation/Leisure activities	High culture	Popular culture	Vocation/Engagement	BurnedOut
*Demographic measures*					
Year of qualification	**-.046 (p = .019) **[**-.046**]	***-.076 (p < .001) ***[***-.078***]	**.046 (p = .025) **[**.050**]	.000 (p = .986) [-.026]	-.004 (p = .827) [.007]
Male	***-.083(p < .001)* [*-.068***]	***.235(p < .001)***[***.220***]	***-.270 (p < .001)***[***-.276***]	.020 (p = .267) [***.076***]	***.089 (p < .001) ***[**.042**]
Number of children	-.023 (p = .368) [***-.152***]	.035 (p = .165) [***-.077***]	***-.116 (p < .001) ***[***-.181***]	.042 (p = .082) **[.064**]	**-.047 (p = .035) **[-.089]
Age of youngest child	***.164 (p < .001)***[***.226***]	***.133 (p < .001) ***[***.162***]	**.*099(p < .001)***[***.177***]	.025 (p = .299) [-.011]	.008 (p = .706) [**.*082***]
*Work variables*					
On Specialist Register	.016 (p = .489) [-.004]	.001 (p = .978) [-.030]	.033 (p = .165) [**.050**]	.028 (p = .202) [.001]	**-.045 (p = .021) **[***-.094***]
On GP register	.030 (p = .266) [.015]	.015 (p = .563) [.035]	.035 (p = .207) [-.034]	**.071 (p = .006)[.057]**	.043 (p = .069) [***.100***]
Number of patients seen	**.044 (p = .042) **[.030]	-.001 (p = .958) [-.024]	***.096(p < .001)***[**.*110***]	***.123 (p < .001) ***[***.150***]	.027 (p = .155) [***.097***]
Sleep deprivation	.000 (p = .985) [-.008]	-.012 (p = .503) [**-.042**]	.022 (p = .239) [***.063***]	-.025 (p = .149) [**-.048**]	***.154 (p < .001) ***[***.277***]
aWPC: Choice and independence	.014 (p = .454) [***.078***]	-.015 (p = .428) [.027]	**.059 (p = .003) **[***.118***]	***.095 (p < .001) ***[***.263***]	***-.097 (p < .001) ***[***-.284***]
aWPC: Workload	-.011 (p = .546) [-.018]	.006 (p = .740) [-.005]	-.035 (p = .063) [-.031]	.011 (p = .542) [-.021]	***.130 (p < .001) ***[***.300***]
aWPC: Supportive-receptive	.025 (p = .157) [**.047**]	.012 (p = .500)[**.038**]	.032 (p = .086) [.027]	***.066 (p < .001) ***[***.194***]	**-.048 (p = .002) **[***-.237***]
aWPL: Deep approach to work	.009 (p = .618) [***.072***]	.022 (p = .206) [***.066***]	-.023 (p = .207) [.031]	***.141 (p < .001) ***[***.255***]	**-.043 (p = .006) **[***-.072***]
aWPL: Surface-rational	***-.089 (p < .001) ***[***-.122***]	***-.089 (p < .001) ***[***-.119***]	-.021 (p = .245) [-.036]	-.005 (p = .767) [-.025]	.022 (p = .135) [**.047**]
aWPL: Surface-disorganised	-.022 (p = .302) [-.032]	-.022 (p = .292)[-.036]	-.005 (p = .818) [.000]	.013 (p = .537) [***-.114***]	.035 (p = .058) [***.241***]
*Personality measures*					
Big 5: Neuroticism	.003 (p = .887) **[-.041]**	-.030 (p = .157) [-.025]	**.051 (p = .023) **[**-.041**]	**-.047 (p = .023) **[***-.251***]	***.326 (p < .001) ***[***.559***]
Big 5: Extraversion	***.136 (p < .001) ***[***.206***]	***.109 (p < .001) ***[***.176***]	***.085 (p < .001)***[***.108***]	***.307 (p < .001) ***[***.457***]	***-.108 (p < .001) ***[***-.315***]
Big 5: Openness to experience	**.210 (p < .001) **[***.310***]	***.261 (p < .001) ***[***.322***]	**-.047 (p = .016) **[**.053**]	**.042 (p = .018) **[***.156***]	**.044 (p = .006) **[.001]
Big 5: Agreeableness	***-.081 <.001) ***[.006]	**-.055 (p = .004) **[***.067***]	***-.069 (p = .001) ***[***-.115***]	.023 (p = .228) [***.145***]	***-.067 (p < .001) ***[***-.220***]
Big 5: Conscientiousness	.007 (p = .741) [**.045**]	.006 (p = .797) [.032]	.005 (p = .826) [.036]	**.092 (p < .001) **[**.244**]	-.020 (p = .314) [**-.252**]
Empathy: Fantasy	***.227 (p < .001) ***[***.295***]	***.187 (p < .001) ***[***.297***]	***.133 (p < .001) ***[***.071***]	.020 (p = .271) [.027]	.019 (p = .238) [**.066**]
Empathy: Perspective-taking	-.007 (p = .710) [***.090***]	-.003 (p = .890) [***.122***]	-.010 (p = .602) [-.040]	.004 (p = .820) [***.100***]	-.026 (p = .123) [***-.131***]
Empathy: Empathic concern	-.019 (p = .286) [**.052**]	-.010 (p = .566) [***.073***]	-.020 (p = .240) [-.027]	.025 (p = .146) [.020]	.015 (p = .341) [***.091***]
Empathy: Personal distress	-.023 (p = .264) [***-.096***]	-.005 (p = .802) [**-.038**]	-.040 (p = .062) [***-.135***]	.030 (p = .131) [***-.203***]	-.009 (p = .622) [***.236***]
Masculinity	-.043 (p = .054) [-.013]	**-.053 (p = .018) **[***-.082***]	.007 (p = .766) [***.130***]	-.010 (p = .627) [***.145***]	**.059 (p = .002) **[***-.135***]
Self-esteem	**.067 (p = .003) **[***.069***]	.048 (p = .029) [.018]	**.052 (p = .024) **[***.115***]	***.118 (p < .001) ***[***.309***]	***-.149 (p < .001) ***[***-.416***]

Note that in the multiple regression, all effects shown take into account all other background measures. For comparison, simple Pearson correlations with each background variable are shown in square brackets in each cell. *Key*: **Bold: p < .05**; **Bold Underlined: p < .01**); **Bold Italicised: p < .001**.

Considering just results with *P *< .001, differences in Avocation/Leisure relate mainly to individual differences (particularly Openness to Experience and the Fantasy component of Empathy, as well as Extraversion, with a smaller negative effect of Agreeableness), and somewhat to demographic factors (with an effect of sex, women having higher scores, and a strong effect of having children, younger children being associated with lower scores); only one of the ten measures relating to work reaches *P *< .001, those with Surface Rational approaches to work having lower scores. The High Culture and Popular Culture broadly show the same patterns of correlations, but with a few important differences. In particular, Openness to Experience relates only (and highly) to High Culture, and there are strong sex differences, women taking part more in High Culture, and men more in Popular Culture.

### Burnout, stress, engagement and satisfaction

Overall 19.2% (541/2,284) of doctors met the conventional criterion for > caseness = of a score of 4 or more on the 0-0-1-1 GHQ-12, which is similar to a previous study [66]. Summary scores were available for stress (GHQ), the three burnout sub-scales (aMBI-EE, aMBI-DP, aMBI-PA), engagement (aUWES) and satisfaction (SATN). The top left portion of Table 4 shows simple correlations between the six scales. The eigenvalues of the correlation matrix were 2.322, 1.382, .853, .573, .487, and .384, and based on a scree-slope analysis, it would appear that there were two factors, which was confirmed by a parallel analysis using 500 boot-strapped replications [96]. Extraction in SPSS used an oblique rotation, since there was no reason to believe the factors were orthogonal, and many theoretical reasons to think that they might be correlated. Oblimin rotation using Kaiser normalization produced the loadings shown in the top right part of the table, the two factors having correlations of -0.203. The first factor, which loaded most heavily on aMBI-EE, and also loaded on GHQ and aMBI-DP, contained many components of stress and burnout and to distinguish it from the raw scale scores was labelled BurnedOut. The second factor loaded almost equally on aUWES and aMBI-PA, and was therefore, following Osler, labelled Vocation/Engagement. The overall satisfaction score (SATN) loaded almost equally on BurnedOut and Vocation/Engagement, so that Satisfaction can be conceptualized as low BurnedOut coupled with high Vocation/Engagement.

**Table 4:** Correlations of stress, burnout and satisfaction measures with leisure and culture scores.

								Factor loadings	
	aMBI-EE	aMBI-DP	GHQ	aMBI-PA	aUWES	SATN		Burned Out	Vocation/ Engagement
aMBI-EE	1.000	***0.367***	***0.408***	-0.043	***-0.062***	***-0.396***		**.862**	.101
aMBI-DP	***0.367***	1.000	***0.150***	***-0.130***	-0.006	***-0.238***		**.663**	.091
GHQ	***0.408***	***0.150***	1.000	***-0.146***	***-0.174***	***-0.340***		**.636**	-.126
aMBI-PA	-0.043	***-0.130***	**-0.146**	1.000	***0.557***	***0.429***		.081	**.872**
aUWES	***-0.062***	-0.006	***-0.174***	***0.557***	1.000	***0.365***		.028	**.869**
SATN	***-0.396***	***-0.238***	***-0.340***	***0.429***	***0.365***	1.000		**-.500**	**.520**
								**Correlations**	
*Avocation/Leisure*	0.007	**-0.044**	***-0.105***	***0.159***	***0.139***	***0.064***		**-0.059**	***0.153***
*High Culture*	0.015	***-0.107***	***-0.072***	***0.130***	***0.119***	0.037		***-0.066***	***0.121***
*Popular Culture*	-0.014	***0.111***	***-0.088***	***0.097***	***0.074***	***0.067***		-0.002	***0.098***

Correlations between the six stress/burnout/engagement/satisfaction scores (top left), and in top right, factor loadings of the scores on the two extracted factors, *Vocation/Engagement *and *BurnedOut *(see text). Bottom left, correlations of the six raw stress/burnout/engagement/satisfaction scores with *Avocation/Leisure activities*, *High Culture *and *Popular Culture*, and bottom right, with *Vocation/Engagement *and *BurnedOut Key*: **Bold: p < .05**; **Bold Underlined: p < .01**); **Bold Italicised: p < .001**.

**Figure 1:**
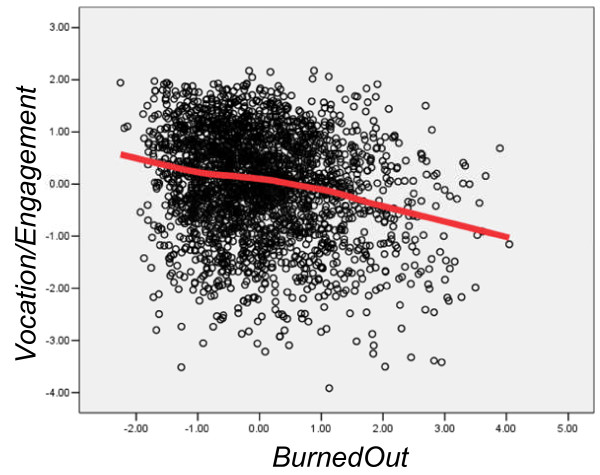
**Scores of individual doctors on Vocation/Engagement (ordinate) and BurnedOut (abscissa)**. The fitted line is a lowess curve. Note that because both measures are factor scores, they are expressed as z-scores (that is, a mean of zero and a standard deviation of one).

Factor scores were extracted using the regression method in SPSS. As reported by Prins *et al*. [72], the correlation between burnout and engagement is relatively weak (r = -.276, *P *< .001), so that there are individuals who are both BurnedOut and have high Vocation/Engagement, and others who are neither BurnedOut nor have high Vocation/Engagement, as can be seen in Figure 1.

### Correlates of BurnedOut and Vocation/Engagement

The right-hand columns of Table 3 show regressions of BurnedOut and Vocation/Engagement on the 25 background factors. BurnedOut related strongly to individual difference variables, particularly to high Neuroticism, but also to low Agreeableness and low Extraversion (as found previously [5]), as well as to low self-esteem. In contrast to the leisure scores, BurnedOut also correlates strongly with work variables, in particular sleep deprivation, high perceived workload, and a lack of choice and independence; men also scored higher. Vocation/Engagement mostly showed a very different pattern of relationships, being associated most strongly with extraversion and conscientiousness, and high self-esteem among the individual difference measures, with a supportive and receptive work environment, a deep approach to work, choice and independence, and seeing more patients. There was no correlation with sex.

Together the 25 background variables accounted for 42.0% of the variance in BurnedOut (R = .648), and 29.6% of the variance in Vocation/Engagement (R = .544). The alpha reliabilities of BurnedOut and Vocation/Engagement were .870 and .829, meaning that 48.3% and 35.7% of the accountable variance in each is explained by the background factors.

### Inter-relations of Avocation/Leisure activities, Vocation/Engagement and BurnedOut

**Figure 2:**
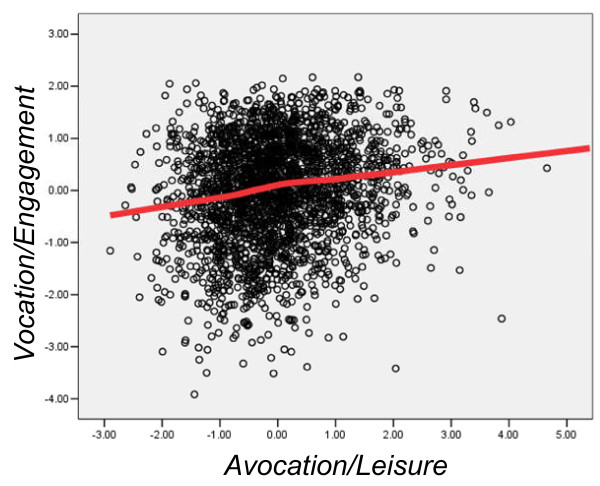
**Scores of individual doctors on Vocation/Engagement (ordinate) and Avocation/Leisure (abscissa)**. The fitted line is a lowess curve. Note that because both measures are factor scores, they are expressed as z-scores (that is, a mean of zero and a standard deviation of one).

**Figure 3:**
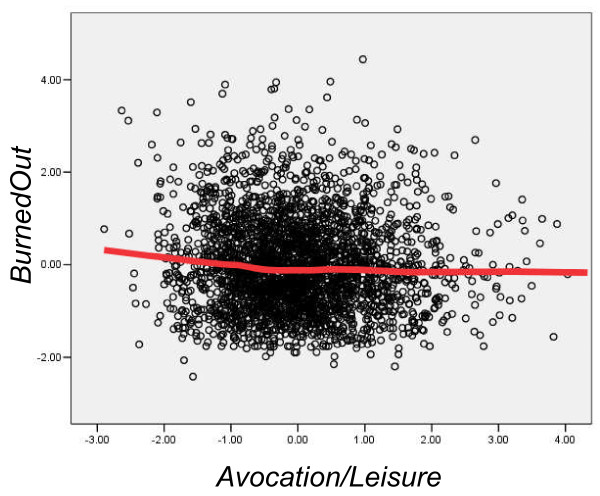
**Scores of individual doctors on BurnedOut (ordinate) and Avocation/Leisure (abscissa)**. The fitted line is a lowess curve. Note that because both measures are factor scores, they are expressed as z-scores (that is, a mean of zero and a standard deviation of one).

In theoretical terms, for testing Osler's hypothesis, the key correlations in Table 4 are in the lower right-hand corner, and show the relationship of Avocation/Leisure with BurnedOut and Vocation/Engagement. These correlations were predicted *a priori *to be the appropriate ones for testing the theory (and indeed, that is why the various measures were included in the study). Avocation/Leisure correlated highly significantly with Vocation/Engagement (R = .170, *P *= 3.03 × 10^-16^) (Figure 2) and the effect remained significant after BurnedOut was taken into account (beta = .161, *P *= 3.23 × 10^-16^). Avocation/Leisure also correlated with BurnedOut (R = -.063, *P *= .0016) (Figure 3) although the effect was no longer significant once Vocation/Engagement was taken into account (beta = -.029, *P *= .143).

The correlation of Avocation/Leisure with Vocation/Engagement was not due to any particular activities, as can be seen in the final column of Table 1, where 20 of the 29 activities correlate positively with Vocation/Engagement, only one correlates negatively (watching television), and the largest correlation is with the very generic, 'Spend time on hobbies'. In general, High Culture and Popular Culture seem to have similar patterns and sizes of correlation with BurnedOut and Vocation/Engagment (Table 4). Both High Culture and Popular Culture continue to correlate significantly with Vocation/Engagement after BurnedOut is partialled out, but do not correlate significantly with BurnedOut after Vocation/Engagement is partialled out. The lower left-hand part of Table 4 also shows the correlations of the three measures of leisure with the six raw scales of stress, burnout, work engagement and satisfaction. In general, these show very similar correlations to those with the factor scores, with the interesting and potentially important exception that the correlations with Depersonalization, which are highly significant and negative with High Culture, are highly significant and positive with Popular Culture (and, perhaps as a result, the correlation with Avocation/Leisure is much smaller and only just reaches the 0.05 level of significance).

## Discussion

The central interest of this paper has been in the suggestion that having a vocation, which many doctors claim to have and can be seen as an integral part of professional behavior, is benefited by having an avocation. Sir William Osler thought that having an avocation would benefit doctors by increasing their sense of vocation and preventing what now we would call burnout, so that he recommended, 'the young doctor should look about early for an avocation, a pastime, that will take him away from patients, pills and potions' [52]. To summarize our results, Osler's ideas are partly supported, in that we find a robust correlation between work engagement and more extensive leisure activities, a result that remained even when twenty-five wide-ranging background variables, including personality, work variables and demographics were taken into account. In contrast, and *contra *Osler, there was no suggestion that leisure activities related to burnout and stress. It is also worth stressing that the measures of leisure, burnout and engagement are robust, with 30.8%, 48.3% and 35.7% of the accountable variance in each being explained by the twenty-five background variables, which is an impressive proportion.

Although Osler talks of 'zeal' (work engagement) and 'the smothering of the flame' (burnout), and implies that they are opposite ends of a single continuum, current research suggests that is not the case. Vocation, which makes medical practice less a job and more an all-consuming passion, is in modern terms 'work engagement', a positive state of work-related well-being characterized by vigor, dedication and absorption [97]; the result is that engaged employees not only have the capacity to be energetic, they enthusiastically apply that energy to their work. They do not hold back. They do not keep their energy in reserve for something important; they become absorbed in their work, experiencing flow in which they lose track of time and diminish their response to distractions [3]. As suggested earlier, the crucial point here is that engagement is seen as the polar opposite of burnout, and some studies have suggested that [98-100]. However, more recent studies have suggested that burnout and engagement are independent constructs [1,101], each having a unique relationship with important factors such as working hours, job characteristics, work outcomes, quality of social relationships, and perceived health [102]. The present study supports that position, not only finding a relatively weak correlation between burnout and engagement, but also finding very different patterns of correlation with other variables, in particular personality, burnout being related to neuroticism (as has been reported in another study elsewhere [103]), and work engagement to extraversion and conscientiousness. As a result doctors can have low levels of work engagement despite not being burnt out. One possible explanation for such a situation might be if a doctor's energy and enthusiasm were focussed outside work, so that work engagement might suffer. However, Sonnentag and colleagues, studying employees in five different industries, found a beneficial effect of disengaging from work when at home [104]. Whether that is true of all individuals is far from clear, and the precise causal relationship between avocations and vocations requires further work.

The data described here are cross-sectional, and therefore cannot prove causality. However, the complete absence of a correlation between Avocation/Leisure and BurnedOut strongly suggests that there can be no causal influence, so that on one prediction based on his writing, Osler was wrong; hobbies do not seem to prevent the flame withering if it is likely to do so. However, on the other side, hobbies do seem to be associated with Osler's sense of 'zeal', described here as Vocation/Engagement, and the correlation remains robust even when a wide range of confounders is taken into account. The direction of causality cannot of course be inferred directly, although it would seem unlikely that higher rates of engagement, which might cause an associated increase in workload, would also result in more leisure activities, so that it is more likely that the causal relation is in the opposite direction. Either way, it might be argued that the association is convincing enough to carry out a longitudinal study or, perhaps better still, a randomized controlled trial, encouraging doctors to increase their avocations, with work engagement as the outcome variable.

Our study also says much about the three main sets of variables: leisure activities, burnout and engagement. Leisure activities in particular have rarely been studied in large cohorts of individuals (although an early attempt was carried out by one of the authors [105]). Leisure activities themselves vary strongly, and it is no surprise to any parent that those with younger children report fewer leisure activities. The strongest correlates of leisure activities we found were with the personality trait of Openness to Experience, and the dimension of empathy known as Fantasy. Interestingly, neither shows a strong relationship to Vocation/Engagement or to BurnedOut, suggesting different underlying processes in their determination. Perhaps of particular importance from the present perspective is that measures related to work, with occasional minor exceptions, show almost no relationships to leisure activities. That suggests that leisure activities are driven to a large extent from within (by personality and by sex), and by the family environment.

Osler was somewhat ambiguous about whether he felt particular avocations to be important. Despite recognizing, 'how absorbing is the profession of medicine', Osler nevertheless advised students that they should 'every day do some reading or work apart from our profession'. Osler was undoubtedly a strong believer in reading being of especial importance (and he quotes Seneca who said, 'If you are fond of books you will escape the ennui of life'). Osler's 'Bed-side library for medical students', was unashamedly literary, containing among others, Shakespeare, Montaigne, Plutarch, Epictetus and *Don Quixote *[106]. He wrote that such reading would enable a student, 'to get the education, if not of a scholar, at least of a gentleman'. Elsewhere Osler is less dogmatic, saying of the particular nature of an avocation, 'I care not what it may be; gardening or farming, literature or history or bibliography...' (although he then does add, 'any one of which will bring you into contact with books'). Nevertheless, the presumption seems to be that what really matters is high culture ('the world of art, of science, or of letters'), an idea reinforced by Sir Geoffrey Keynes in his first Oslerian Lecture [107], where he said, '[Osler] believed that 'culture' ... was of the utmost value to medical men' (and that concept is also found in writers such as C. P. Snow, who said there ought to be 'a literary component through the course of medical education' [108]). That can also be seen when Osler writes, '... it makes precious little difference what the outside interest may be ... [but] I would like to make a plea for the book' [52 p.927]. Factor analysis of our list of leisure activities (as elsewhere [82,105]) clearly shows a split into what we have called High Culture and Popular Culture (and those two factors also emerged in a much earlier study of medical students by one of us [105]). However, and it is a key interpretative finding, although High Culture shows a slightly higher correlation with Vocation/Engagement than does Popular Culture, both measures show very significant correlations. In analyses not reported here, it was also the case that no particular subset of the 29 activities particularly correlated with Vocation/Engagement (see the last column of Table 1). As far as the beneficial effect of any particular vocation is concerned, Osler was perhaps more right to say, 'I care not what it may be'.

One intriguing result in table 4, is that although five of the six measures of stress/burnout/engagement and satisfaction correlate in very similar ways with the avocation and leisure methods, an exception is that Depersonalization correlates significantly and negatively with High Culture, whereas it correlates significantly and positively with Popular Culture (and, perhaps as a result, the correlation with Avocation/Leisure is much smaller and only just reaches the 0.05 level of significance). Whether these differences are meaningful is not clear, although it is possible that emotional exhaustion and depersonalization are different psychological states, as is suggested by the fact that they may have different causal relations with one another in men and women [109], they can have different correlations with job-related measures [110], and perhaps also have different correlates with personality (for example, EE has been said to correlate mainly with neuroticism, whereas DP correlates mainly with lower extraversion, lower agreeableness and lower openness to experience [111], correlations which, it must be said, are not entirely replicated in the presented data). It is a possibility, though, that popular culture is depersonalizing, treating people as things, whereas high culture encourages the opposite, treating people as individuals.

Further investigations are needed to identify the mechanisms or processes by which Avocations/Leisure activities relate to higher levels of Vocation/Engagement. One possibility is that leisure activities, like all cultural activities, vicariously increase a person's knowledge of the world, be it the physical world and its geography and environment, the social world, with its historical and geographical differences, or the interpersonal world, with its complex emotional interactions between people with different needs [47,112]. Avocations, in other words, increase cultural capital, and that capital can then be used to advantage within the professional engagements of a medical career. An alternative possibility is that leisure activities result in positive mood states [113] precisely because it is the person himself or herself who has chosen those activities, and they are under personal control (and then the particular avocation itself would matter little). To finish with Osler's words, it may be precisely the 'selection and the choice' which are important, a selection and choice which are entirely under the control of the doctor and the doctor alone, and hence 'will be ... according to [the doctor's] tastes'.

## Conclusions

Doctors differ in the extent of their hobbies and non-medical activities (Avocation/Leisure), as also in whether they are BurnedOut or show Vocation/Engagement, each measure having a high proportion of its total variance related to background variables, and each showing different patterns of correlation with demographic, work, and personality measures. Avocation/Leisure correlated with Vocation/Engagement but not with BurnedOut, which has important implications, not only for understanding the nature of burnout and work engagement, which increasingly appear to be separate states, but also in improving the sense of engagement in doctors.

## List of abbreviations

aAWQ: Abbreviated Approaches to Work Questionnaire; aMBI: Abbreviated Maslach Burnout Inventory; aUWES: Abbreviated Utrecht Work Engagement Scale; aWCQ: Abbreviated Workplace Climate Questionnaire; AWQ: Approaches to Work Questionnaire; DP: Depersonalization; EE: Emotional exhaustion; GHQ: General Health Questionnaire; GMC: General Medical Council; GP: General Practitioner; LRMP: List of Registered Medical Practitioners; MBI: Maslach Burnout Inventory; Q: Questionnaire numbering; R: Multiple correlation coefficient; PA: Personal accomplishment; SATN: Satisfaction with medicine as a career; UCL: University College London; UK: United Kingdom; UWES: Utrecht Work Engagement Scale; WCQ: Workplace Climate Questionnaire.

## Competing interests

The authors declare that they have no competing interests.

## Authors' contributions

The studies of medical student selection and training were begun by ICM and PR, and EP collaborated in subsequent follow-ups. ICM designed the present follow-ups, which were carried out on a day-to-day basis by HJ. All authors contributed to the analysis and interpretation of the results. The first draft of the manuscript was written by ICM, and all authors contributed to the final version of the manuscript.

## Pre-publication history

The pre-publication history for this paper can be accessed here:

http://www.biomedcentral.com/1741-7015/9/100/prepub

## Supplementary Material

Additional file 1 **Details of the questionnaire used in the study**. Supplementary file containing details of the questionnaire. Click here for file
