# Supplementary material for: Vocation and avocation: leisure activities correlate with professional engagement, but not burnout, in a cross-sectional survey of UK doctors
Source: BMC Med. 2011 Aug 30;9:100. doi: 10.1186/1741-7015-9-100 (PMC3196901; doi:10.1186/1741-7015-9-100)
Supplement: Additional file 1 — Details of the questionnaire used in the study. Supplementary file containing details of the questionnaire. [file 1741-7015-9-100-S1.PDF]

Additional File 1

The questionnaire that was sent to the doctors in the survey. This particular version is for the 1991 cohort (who had applied to medical school in 1990), but the others are almost identical except for question 2, which refers to different years for the career summary. Note that some items for question 17 have been removed due to possible copyright restrictions. Not all questions asked in the study have been referred to in the present paper but are for other purposes.

1990 Medical School Applicants: 2009 follow-up

1. **How would you describe your current job?** If not currently employed, tick here ☐ and describe your most recent post.

	Level	*Speciality*	*Sessions (half-days) per week*	*Since when have you been in this post?*	*Is this an academic post?*
**Main Medical post**					
**Subsidiary Medical post(s)**					
**Non-medical post**					

IF YOU ARE **ENTIRELY IN A NON-MEDICAL JOB,** PLEASE NEVERTHELESS COMPLETE AS MANY QUESTIONS AS POSSIBLE.

2. **Please describe the course of your career from 1995 until the present day. For each year put a tick to indicate the post you held. Do not worry about being too precise** (for instance, if your PRHO posts were from Aug 96 until Jul 97 then just put a tick under 96). **If you were in several categories within a year then just tick them all. If you were part-time, indicate that in the row provided**.

	1995	1996	1997	1998	1999	2000	2001	2002	2003	2004	2005	2006	2007	2008
Student														
PRHO														
SHO														
Registrar														
Senior Registrar														
Specialist Registrar														
Clinical Fellow														
Associate Specialist														
Consultant														
Other Hospital Grades														
GP Trainee / Registrar														
GP Assistant														
GP Principal														
Salaried GP														
Other Medical														
Other non-Medical														
Researcher														
Lecturer														
Senior Lecturer														
Reader														
Professor														
Not working														
If ***part-time***, tick here														

3. **What post-graduate examinations such as Degrees, Memberships, Fellowships, Diplomas, and Certificates have you obtained?** For Memberships or Fellowships taken in several parts, please describe each part separately.

Qualification	*Part*	*Year*	*Qualification*	*Part*	*Year*	*Qualification*	*Part*	*Year*

4. **Have you been awarded a CCST or CCT ?** *No / Yes* **If *Yes*, when was it awarded?** ________________________
If *No*, do you have a CCST or CCT date? *No / Yes* ________

5. **How many patients do you see *in a typical week*?**
Emergency admissions _______ *Routine admissions* ________ *Day care* ________
Out-Patient Department _______ *General Practice* _________ *Other* ________

6. **How often does your typical work schedule leave you feeling short of sleep when at work?**
Daily / Several times a week / Weekly / Several times a month / Monthly / Rarely / Never

7. **Are you involved in clinical audit?** *I am leading on one or more audits / I am helping with one or more audits / I have been to audit meetings / I know it is going on / Not at all*

8. **Are you currently undertaking any work-related leadership roles in addition to your normal clinical role** (e.g. clinical directorate, quality committee, training programme, etc..) **?** *No / Yes*
If *Yes*:

What are the roles?	
What benefits do you get from them?	

What are their disadvantages?	

9. **Are you currently:** *Single / Married-Cohabiting / Divorced / Widowed ?*

If *Married-Cohabiting*, is your partner: **Qualified as a doctor?** *No / Yes*
Qualified as a non-medical health-care professional? *No / Yes*
Working at present: *Full-time / Part-time / Not working*

10. **How many children do you have? _____ What years were they born? ___________________________________**

11. **Do you have a research degree or are you preparing for one?**

☐ *Yes.* **If so, is it a** *PhD / MD / Other* __________ ? **What date was it conferred?** ______
☐ *I am currently registered for a research degree.* **If so, is it a** *PhD / MD / Other* __________ ?
☐ *No, but I would like to take a research degree in the future.*
☐ *No, and I have no intention of taking one in the future*

12. **How many peer-reviewed research papers have you published?** *None / 1-2 / 3-5 / 6-10 / 11-20 / 21-50 / 51-100 / 101+*

13. **Did you publish any peer-reviewed research papers as an undergraduate?** *None / 1-2 / 3-5 / 6+*

14. **Irrespective of your current career post, <u>if you were starting a medical career again</u>, how attractive would you find each of these *eleven broad areas of medical practice* as a speciality?**

	Extremely attractive	*Very attractive*	*Fairly attractive*	*Fairly unattractive*	*Very unattractive*
Anaesthetics					
General Practice					
Hospital medicine					
Medical administration					
Obstetrics and Gynaecology					
Pathology (inc. Microbiology, Haematology, etc.)					
Paediatrics & Child Health					
Psychiatry / Mental health					
Public Health / Community Medicine					
Radiology / Radiotherapy					
Research					
Surgery (inc A&E and Ophthalmology)					

15. **How well do the following statements describe your working environment?**

	Definitely disagree	*Somewhat disagree*	*Somewhat agree*	*Definitely agree*
i. At work I find it difficult to organise my time effectively				
ii. When I have something to do at work, I like to know precisely what is expected				
iii. Some of the issues that crop up at work are so interesting that I pursue them though they are not part of my job				
iv. My habit of putting off work leaves me with far too much catching up to do				
v. I prefer the work I am doing to be clearly structured				
vi. I spend a good deal of my spare time learning about things related to my work				
vii. There is a real opportunity in my job for me to choose the particular things I work on				
viii. My work colleagues really try hard to get to know one another				
ix. I have a lot of choice about the work I do				
x. My job requires me to do too many different things				
xi. My coworkers are supportive and friendly towards me				
xii. There seems to be too much work to get through in my job				

16. **How often do the following statements describe the way you feel about working as a doctor?**

	Every day	*A few times a week*	*Once a week*	*A few times a month*	*Once a month or less*	*A few times a year*	*Never*
i. I deal very effectively with the problems of my patients							
ii. I feel I treat some patients as if they were impersonal objects							
iii. I am proud of the work that I do							
iv. I feel emotionally drained from my work							
v. I feel fatigued when I get up in the morning and have to face another day on the job							
vi. At work I feel bursting with energy							
vii. I've become more callous towards people since I took this job							
viii. I feel I'm positively influencing other people's lives through my work							
ix. Working with people all day is really a strain for me							
x. When I am working I forget everything else around me							
xi. I don't really care what happens to some patients							
xii. I feel exhilarated after working closely with my patients							
xiii. I think of giving up medicine for another career							

xiv. I reflect on the satisfaction I get from being a doctor							
xv. I regret my decision to have become a doctor							

17. Do you agree with the way these statements describe you as a person?

	Strongly Disagree	*Disagree*	*Neutral*	*Agree*	*Strongly agree*
xviii. When people compliment me I sometimes get embarrassed					
xix. I expect a great deal from other people					
xx. I insist upon getting the respect that is due to me					
xxi. There is a lot that I can learn from other people					
xxii. I find it difficult to depend on other people					
xxiii. I find it easy to get emotionally close to others					
xxiv. I prefer not to have other people depend on me					
xxv. I worry that others don't value me as much as I value them					
xxvi. I am quick to spot when someone in a group is feeling uncomfortable					
xxvii. I am fascinated by how machines work					
xxviii. I find it hard to know what to do in a social situation					
xxix. I do not enjoy games that involve a high degree of strategy					

18. Please indicate how well each of the following statements describes you:

	Describes me very badly	*Describes me poorly*	*Describes me fairly well*	*Describes me very well*
i. I daydream and fantasise with some regularity about things that might happen to me				
ii. I sometimes find it difficult to see things from another person's point of view				
iii. Sometimes I don't feel very sorry for other people when they are having problems				
iv. In emergency situations I feel apprehensive and ill-at-ease				
v. I try to look at everybody's side of a disagreement before I make a decision				
vi. Becoming extremely involved in a good book or movie is somewhat rare for me				
vii. Other people's misfortunes do not usually disturb me a great deal				
viii. Being in a tense, emotional situation scares me				
ix. I am usually pretty effective in dealing with emergencies				
x. I would describe myself as a pretty soft-hearted person				
xi. I really get involved with the feelings of the characters in a novel				
xii. Before criticising somebody I try to imagine how I would feel if I were in their place				
xiii. I'm always willing to admit it when I make a mistake				
xiv. No matter who I'm talking to, I'm always a good listener				
xv. I sometimes feel resentful when I don't get my way				
xvi. I am able to do things as well as most other people				
xvii. I feel useless at times				
xviii. I take a positive attitude towards myself				
xix. At times I am inclined to feel that I am a failure				

19. Each pair of phrases describes two behaviours. Each of us is somewhere between the two extremes. Put a mark between the two to best describe yourself.

	+++	++	+	-	+	++	+++	
Not at all independent								Very independent
Not at all emotional								Very emotional
Very rough								Very gentle
Not at all competitive								Very competitive
Not at all kind								Very kind
Not at all aware of feelings of others								Very aware of feelings of others
Gives up very easily								Never gives up easily
Not at all self confident								Very self confident

20. How has your health been in general *over the past few weeks*. Have you recently:

Been able to concentrate on whatever you're doing?	*Better than usual*	*Same as usual*	*Less than usual*	*Much less than usual*
Lost much sleep over worry?	*Not at all*	*No more than usual*	*Rather more than usual*	*Much more than usual*
Felt that you were playing a useful part in things?	*More so than usual*	*Same as usual*	*Less useful than usual*	*Much less useful*
Felt capable of making decisions about things?	*More so than usual*	*Same as usual*	*Less so than usual*	*Much less capable*
Felt constantly under strain?	*Not at all*	*No more than usual*	*Rather more than usual*	*Much more than usual*
Felt that you couldn't overcome your difficulties?	*Not at all*	*No more than usual*	*Rather more than usual*	*Much more than usual*
Been able to enjoy your normal day-to-day activities?	*More so than usual*	*Same as usual*	*Less so than usual*	*Much less than usual*
Been able to face up to your problems?	*More so than usual*	*Same as usual*	*Less able than usual*	*Much less able*
Been feeling unhappy and depressed?	*Not at all*	*No more than usual*	*Rather more than usual*	*Much more than usual*
Been losing confidence in yourself?	*Not at all*	*No more than usual*	*Rather more than usual*	*Much more than usual*
Been thinking of yourself as a worthless person?	*Not at all*	*No more than usual*	*Rather more than usual*	*Much more than usual*
Been feeling reasonably happy, all things considered?	*More so than usual*	*About same as usual*	*Less so than usual*	*Much less than usual*

21. Please describe a little about your interests and activities outside your job.

How often do you?

	Every day	*A few times a week*	*Once a week*	*A few times a month*	*Once a month or less*	*A few times a year*	*Never*
Listen to popular music							
Listen to classical music							
Go to pop concerts / discos							
Go to classical music concerts / opera							
Play a musical instrument							
Go to museums or art galleries							
Read about art in newspapers, magazines or books							
Draw, paint, sculpt or do other arts or crafts							
Photography							
Read a novel							
Read non-fiction books (not for work or study)							
Read poetry							
Write poetry, fiction or other literature (not for work)							
Go to the cinema							
Go to the theatre (plays/musicals, etc)							
Acting or otherwise taking part in theatre							
Watching classical or modern ballet/dance							
Dance (any form)							
Play sport							
Watch sport							
Hike / Orienteer / Climb / Mountaineer / Ski etc.							
Cook							
Shop (for pleasure)							
Spend time on hobbies (excluding above activities)							

	--------------------- Most days for -------------------- 4+ hours	*2-4 hours*	*1-2 hours*	*1 hour or less*	*2-3 times a week*	*Once a week*	*Less often*
Watch television							
Watch DVDs / videos / etc..							
Listen to radio							
Listen to podcasts							
Browse the internet (not for work)							

22. How long did it take you to complete this questionnaire? ___________*minutes*

Thank you for completing this detailed questionnaire and helping with the survey

This questionnaire is entirely for the purposes of research. Both for your own reassurance and as a normal part of research ethics, we would be grateful if you would **acknowledge the declaration by initialling it.**

DECLARATION: *This questionnaire is entirely for the purposes of educational research, its contents will be kept strictly confidential, will not be made known to anyone outside of the research study, and will not otherwise be disclosed or published except in an aggregated form in which individuals cannot be identified.*

I C McManus MD PhD FRCP FRCPEd FMedSci

Please acknowledge by initialling:

______(Initials) ___/____/200____
